# Glycogen synthase kinase-3 inhibition disrupts nuclear factor-kappaB activity in pancreatic cancer, but fails to sensitize to gemcitabine chemotherapy

**DOI:** 10.1186/1471-2407-9-132

**Published:** 2009-04-30

**Authors:** Shadi Mamaghani, Satish Patel, David W Hedley

**Affiliations:** 1 Division of Applied Molecular Oncology, University Avenue, Toronto, Ontario, Canada; 2 Department of Laboratory Medicine and Pathobiology, University of Toronto, Toronto, Ontario, Canada; 3 Princess Margaret Hospital, University Avenue, Toronto, Ontario, Canada; 4 Samuel Lunenfeld Research Institute, Mount Sinai Hospital, 600 University Avenue, Toronto, Canada; 5 Department of Medical Oncology and Hematology, Princess Margaret Hospital, 610 University Avenue, Toronto M5G 2M9, Canada

## Abstract

**Background:**

Aberrant activation NF-kappaB has been proposed as a mechanism of drug resistance in pancreatic cancer. Recently, inhibition of glycogen synthase kinase-3 has been shown to exert anti-tumor effects on pancreatic cancer cells by suppressing NF-kappaB. Consequently, we investigated whether inhibition of GSK-3 sensitizes pancreatic cancer cells to the chemotherapeutic agent gemcitabine.

**Methods:**

GSK-3 inhibition was achieved using the pharmacological agent AR-A014418 or siRNA against GSK-3 alpha and beta isoforms. Cytotoxicity was measured using a Sulphorhodamine B assay and clonogenic survival following exposure of six different pancreatic cancer cell lines to a range of doses of either gemcitabine, AR-A014418 or both for 24, 48 and 72 h. We measured protein expression levels by immunoblotting. Basal and TNF-alpha induced activity of NF-kappaB was assessed using a luciferase reporter assay in the presence or absence of GSK-3 inhibition.

**Results:**

GSK-3 inhibition reduced both basal and TNF-alpha induced NF-kappaB luciferase activity. Knockdown of GSK-3 beta reduced nuclear factor kappa B luciferase activity to a greater extent than GSK-3 alpha, and the greatest effect was seen with dual knockdown of both GSK-3 isoforms. GSK-3 inhibition also resulted in reduction of the NF-kappaB target proteins XIAP, Bcl-X_L_, and cyclin D1, associated with growth inhibition and decreased clonogenic survival. In all cell lines, treatment with either AR-A014418, or gemcitabine led to growth inhibition in a dose- and time-dependent manner. However, with the exception of  PANC-1 where drug synergy occurred with some dose schedules, the inhibitory effect of  combined drug treatment was additive, sub-additive, or even antagonistic.

**Conclusion:**

GSK-3 inhibition has anticancer effects against pancreatic cancer cells with a range of genetic backgrounds associated with disruption of NF-kappaB, but does not significantly sensitize these cells to the standard chemotherapy agent gemcitabine. This lack of synergy might be context or cell line dependent, but could also be explained on the basis that although NF-kappaB is an important mediator of pancreatic cancer cell survival, it plays a minor role in gemcitabine resistance. Further work is needed to understand the mechanisms of this effect, including the potential for rational combination of GSK3 inhibitors with other targeted agents for the treatment of pancreatic cancer.

## Background

Surgery is the only curative treatment for pancreatic cancer, but the majority of patients have metastatic disease or an unresectable tumor at diagnosis [1,2]. Due to the poor response to chemo- and radiation therapies, the disease is highly lethal [2]. Gemcitabine (difluorodeoxycytidine) is the most active chemotherapy agent used for the treatment of pancreatic cancer [3]. It is an analog of deoxycytidine, that gets incorporated into double stranded DNA during S phase, resulting in inhibition of DNA synthesis, arrest of the cell cycle progression, and induction of apoptosis [4]. However, due to pre-existing or acquired chemoresistance, gemcitabine treatment has a marginal survival benefit and yields an objective tumor response rate of < 10% [5,6].

Multiple lines of evidence suggest that aberrantly activated nuclear factor-kappa B (NF-κB) plays a major role in metastasis, cell proliferation, angiogenesis, and chemotherapy resistance of several tumor types including pancreatic cancer [7-11]. Activated NF-κB has been observed in pancreatic cancer cell lines and animal models of pancreatic cancer, as well as primary human pancreatic cancers [7,12,13].

The NF-κB family of transcription factors [p65, p50, p52, RelB, and c-Rel] is involved in the activation of a broad range of genes involved in inflammation, differentiation, tumourigenesis, metastasis, embryonic development, and apoptosis [11,12,14]. They are activated in response to extracellular stimuli including inflammatory cytokines and growth factors, which results in the phosphorylation and subsequent degradation of the NF-κB inhibitor IκB. Additional levels of NF-κB regulation include phosphorylation of p65 at various sites, although these are less well characterized. NF-κB target genes encode cytokines [IL-1, IL-12, IL-2, IL-6, IL-8, IL-10, TNF-α, interferon-β], transcription factors [c-Myc], inhibitors of apoptosis [Bcl-2, Bcl-X_L_, XIAP, FLIP], mitogenic factors [cyclin D1], and cell adhesion molecules [E-selectin, ICAM-1, VCAM-1] [15-17]. Previous *in vitro *studies have shown that inhibition of NF-κB using IκBα super-repressor or sulfasalizine enhances the effect of chemotherapeutic agents in pancreatic cancer cell lines [18,19]. Furthermore, inhibition of NF-κB by the natural compound curcumin was reported to potentiate the antitumor activity of gemcitabine in an orthotopic xenograft model of pancreatic cancer [20]. Together, these findings suggest that aberrant activation of NF-κB leads to chemoresistance in pancreatic cancer, and that inhibition of NF-κB sensitizes the treatment outcome.

Glycogen synthase kinase-3 (GSK-3) is a constitutively active serine-threonine kinase that can phosphorylate and inactivate a broad range of substrates including glycogen synthase, cyclin D1, Mcl-1, c-myc, c-jun, β-catenin, tau, notch, and HIF-1 [21]. Mammalian GSK-3 exists as two isoforms, α and β, with semi-redundant actions that are ubiquitously expressed in tissues [21,22]. *In vivo *and *in vitro *studies have shown that GSK-3 can phosphorylate and regulate NF-κB in a dual mode. The p65 subunit of NF-κB has been reported to be phosphorylated by GSK-3 at serine 468 resulting in its decreased activity [23]. Nonetheless, mice engineered to lack both GSK-3β alleles are sensitive to TNF-α and die in late gestation due to massive liver apoptosis; a phenotype similar to mice lacking p65 subunit of NF-κB or IKKβ [24,25]. Hepatocytes pretreated with a GSK-3 inhibitor LiCl, were also shown to have lower NF-κB activity, as measured by NF-κB dependent luciferase assay. Furthermore, mouse embryonic fibroblasts (MEFs) deficient in both alleles of GSK-3β fail to activate NF-κB after treatment with TNF-α, when compared to wild type MEF [26]. Pharmacological or siRNA mediated inhibition of GSK-3β has been shown to reduce NF-κB mediated gene transcription and inhibit the growth of cancers that show high NF-κB activity including pancreatic cancer [8,27,28]. These results point to a possible role for GSK-3 in the maintenance of high NF-κB activity in cancer cells. Since aberrant NF-κB activation has been linked to drug resistance in pancreatic cancer, we tested the hypothesis that reduction of NF-κB activity through GSK-3 inhibition sensitizes pancreatic cancer cells to chemotherapy.

## Methods

### Reagents and antibodies

Curcumin (Diferulylmethane, 80% pure; 98% curcuminoid content), was obtained from Sigma-Aldrich Canada Ltd. (Oakville, Ontario, Canada), and GSK-3 Inhibitor VIII [AR-A014418 (AR-18)] was obtained from CALBIOCHEM^®^, EMD Biosciences, Inc. (San Diego, CA). Both agents were dissolved in DMSO and aliquots stored at -20°C. Gemcitabine from Eli Lilly (Indianapolis, IN) was freshly prepared as 10 mM stock in sterile PBS on the day of use.

Rabbit polyclonal antibodies against XIAP, β-catenin, and Bcl-X_L _were purchased from Cell Signaling Technology (Danvers, MA). Rabbit monoclonal cyclin D1 antibody was obtained from Lab Vision Corp. (Fremont, CA). A mouse monoclonal antibody against GSK-3 α/β was obtained from Biosource Inc. (Camarillo, CA). Anti-rabbit and anti-mouse horseradish peroxidase linked IgG antibodies, were from Amersham Biosciences (Buckinghamshire, United Kingdom). Recombinant Human TNF-α/TNFSF1A was purchased from R&D Systems (Minneapolis, MN)

### Cell lines and media

The pancreatic cancer cell lines BxPC-3, MIA PaCa-2, PANC-1, and HPAC were obtained from the American Type Culture Collection (Rockville, MD), and PK-1 and PK-8 were from Dr. Masao Kobari (Sendai, Japan). BxPC-3, PK-1, and PK-8 cell lines were cultured in RPMI 1640. PANC-1 and MIA PaCa-2 cell lines were cultured in Dulbecco Eagles medium. HPAC cells were cultured in HAM F-12. All the media for cell culture were supplemented with 10% fetal bovine serum (FBS), 100 units/mL penicillin and 100 μg/mL streptomycin, and cells were grown at 37°C and 5% CO_2 _in air. Additional 2.5% horse serum was added to the media growing MIA PaCa-2 cells.

### Cell treatments, lysate preparation, and immunoblotting

Cells grown at 60% to 70% confluency were exposed to different doses of AR-18 (0–50 μM), or lithium chloride (LiCl) (0–50 mM), potassium chloride (KCl) (10 mM), or solvent control, and incubated at 37°C in a CO_2 _incubator. After 48 h, the cells were lysed using RIPA buffer [20 mM Tris (pH 7.5), 150 mM NaCl, 1% NP-40, 0.5% Na deoxycholate, 0.1% SDS, and 1 mM EDTA supplemented with 1 mM Na_3_VO_4_, protease inhibitor cocktail (Roche Diagnostics) and a serine/threonine-phosphatase inhibitor cocktail 1 (Sigma-Aldrich). Alternatively, drug treated cells were lysed and fractionated to separate the cytoplasmic content using hypotonic lysis buffer [50 mM Tris (pH 7.4), 1 mM EDTA, 10 mM NaF, 1 mM Na_3_VO_4 _and supplemented with protease inhibitor cocktail (Roche Diagnostics) and serine/threonine-phosphatase inhibitor cocktail 1 (Sigma-Aldrich)]. The protein content of the supernatants was measured using bicinchoninic acid protein assay from Pierce PerBio (Rockford, IL) and twenty five micrograms of the lysates were resolved on 8% or 10% SDS-PAGE gels. The resolved proteins were transferred onto polyvinylidene difluoride membranes (Millipore, Bedford, MA), blocked with 5% non-fat milk, and probed with the appropriate antibodies according to the manufacturer's recommendation. The blots were washed, and exposed to the appropriate HRP-conjugated secondary antibodies for 1 h at room temperature. Detection was done using SuperSignal^® ^West Pico from Pierce BioLynx Inc. (Brockville, Ontario, Canada) reagent or enhanced chemiluminescence plus (ECL Plus) kit (Amersham Biosciences). Cytoplasmic lysates were used for detection of β-catenin, whereas the rest of the proteins were detected using the RIPA lysates. Blotting for α-tubulin from Oncogene Research Products, Calbiochem, (San Diego, CA) or β-actin from Abcam Antibodis, Inc., (Cambridge, MA) were used to control for protein loading.

### Proliferation assay

The effect of AR-18, gemcitabine, and curcumin on cell proliferation was determined by the Sulphorhodamine B (SRB) dye (Molecular Probes, Eugene, OR) binding assay as described previously [29]. Briefly, 5,000 cells per well were seeded in 96-well plates, incubated in a CO_2 _incubator overnight at 37°C, and then treated with different doses of curcumin (0–50 μM), AR-18 (0–50 μM) or gemcitabine (0–1 μM) alone or in combination (i.e. concurrent or sequential) in triplicates for 24, 48, and 72 h. The cells were then fixed using 10% (v/v) trichloroacetic acid for 1 h at 4°C, washed extensively with water, stained with 0.4% SRB dissolved in 1% (v/v) acetic acid in water reagent for 30 minutes at room temperature, and then washed, and 10 mM unbuffered Tris was added to each well. The absorbance was measured at 570 nm using a Multiscan 96-well plate reader from Thermo Electron Corp. (Milford, MA). This experiment was repeated three times in six replicates.

### Transient transfection and luciferase assay

PANC-1, MIA PaCa-2, PK-1, and PK-8 cells were seeded in 12-well plates (130,000 per well) in antibiotic-free medium containing 10% FBS. The cells were incubated in a CO_2 _incubator overnight at 37°C prior to transfection using Lipofectamine 2000 from Invitrogen Life Technologies,(Carlsbad, CA) as recommended by the manufacture. Briefly, 0.5 μg/well TA-LUC NF-κB (from Dr. T. Pawson, Samuel Lunenfeld Research Institute, University of Toronto), and 0.05 μg/well β-gal CMV (from Dr. W.C. Yeh, Ontario Cancer Institute, University of Toronto) were co-transfected to the cells. After 16 h, the medium was changed and the cells were incubated with AR-18 (50 μM), gemcitabine (10 μM), or curcumin (50 μM) alone or in combination for 8 h. TNF-α (30 ng/mL) was added to the cells 4 h prior to cell lysis. Control cells were transfected with the plasmids, but did not receive any drug treatments. Luciferase activity was measured by using the Dual-Light^® ^System luciferase assay from Applied Biosystems (Bedford, MA) according to the manufacturer's protocol. The luminometer used was Luminoskan Ascent from ThermoLab Systems (Franklin, MA). The results were normalized to the values read for β-galactosidase activity. All experiments were performed in triplicate and were repeated four times.

### Genetic knockdown of GSK-3

PANC-1 cells were transfected using a reverse transfection protocol. Briefly, the cells were seeded at 300,000 cells per well in 6-well plates, then placed in a CO_2 _incubator at 37°C for 1 h prior to transfection with either silencer negative control siRNA or anti-GSK-3β from Applied Biosystems, Ambion Inc. (Bedford, MA), or anti-GSK-3α [Hs_GSK3A_5_HO Validated] from Qiagen, Inc. (Mississauga, Ontario, Canada) or both by using Hiperfect transfection reagent from Qiagen Inc. according to the manufacturer's protocol. After 72 h, the cells were lysed using RIPA or hypotonic lysis buffers and the proteins present in cell lysates were resolved in SDS-PAGE. Preliminary experiments showed that the concentrations of siRNA required achieving >80% knockdown of GSK3α and GSK3β were 10 nM and 80 nM, respectively. These concentrations were used in all the studies.

To combine the luciferase assay with genetic knockdown of GSK-3, 24 h after siRNA transfection, the medium was changed and the cells were subjected to co-transfection with TA-LUC NF-κB and β-gal CMV as previously described. After 24 h exposure, the medium was changed and the cells were incubated for 48 h prior to exposure to rather TNF-α (30 ng/mL, 4 h) or gemcitabine (10 μM, 8 h). Subsequently, the cells were lysed and the whole cell lysates were used for luciferase assay as described above.

### Clonogenic assay

The effect of AR-18 and of gemcitabine on survival of PANC-1 and BxPC-3 cells was further investigated by a colony-forming assay as described by Wu *et al. *[30]. In brief, exponentially growing cells were treated with either gemcitabine (0.001–10 μM), AR-18 (10–50 μM), or both for 24 h. The cells were then trypsinized and washed twice with PBS to remove the remaining drug, counted, and then seeded in 10× and 5× serial dilutions for PANC-1 and BxPC-3 cells respectively. The plates were incubated for 16 days at 37°C in a CO_2 _incubator at 90% humidity. The plates were then stained with methylene blue from Fischer Scientific, (Ottawa, ON) and colonies were counted. The experiments were performed in triplicates, and at least three times for each cell line.

### Statistical analysis

All the statistical analysis was performed by the help of "R" software (Hornik *et al*.; http://www.r-project.org). To investigate the possible synergistic effect of combining two agents, the interaction between the two drug treatments was tested by fitting it into a model that considers the fact that some experiments were not performed at the same time. The values of optical density (for SRB), colony count (clonogenic assay), or luciferase unit (luciferase assay) were log transformed to stabilize the variance of the residuals. The resulting values were analysed by comparing between different concentrations of each drug using linear regression models. A drug interaction was considered synergistic when the effect of the drug combination was significantly greater than the sum of the effects of both drugs, and sub-additive when it was less than that.

## Results

### Proliferation and colony-forming capacity of pancreatic cancer cells is decreased after pharmacological inhibition of GSK-3

**Figure 1:**
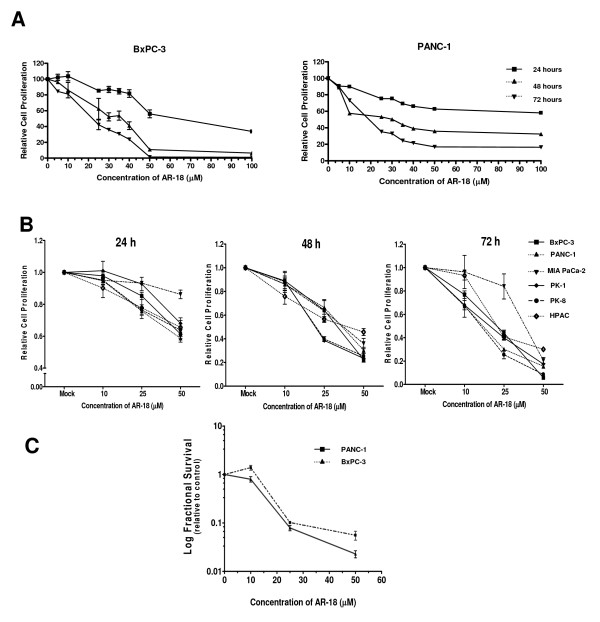
**Inhibition of GSK-3 decreases proliferation and clonogenic survival of pancreatic cancer cells in a dose- and time-dependent manner**. **A**. Effects of AR-18 (μM) on the growth inhibition of BxPC-3 and PANC-1 cells after 24, 48, and 72 h of drug exposure measured by SRB assay. Each point signifies mean from three experiments, each including six replicates; error bars = ± SEM. The results are relative to untreated control. **B**. Growth inhibitory effect of AR-18 (μM) against six pancreatic cancer cell lines after exposure for 24, 48, and 72 h, measured by SRB assay. Each point signifies mean from three separate experiments, each including six replicates; error bars = ± SEM. The results are relative to untreated control. **C**. Effects of AR-18 on the number of colony-forming PANC-1 and BxPC-3 cells after drug exposure for 24 h. Control cells were given vehicle solution. Each point represents mean for four experiments, each containing three replicates; error bars = ± SEM. The results are relative to untreated control.

Consistent with previous reports [28], treatment of PANC-1 and BxPC-3 cells with the GSK-3 inhibitor AR-18 caused a growth inhibitory effect in a dose- and time-dependent manner. Depending on the duration of exposure, the IC_50 _values ranged from as low as 20 μM to as high as 65 μM. After 48 h exposure, the (IC_50_) of AR-18 was approximately 30 μM for both cell lines (Fig. 1A). A range of AR-18 doses below and above this range was used for all our experiments, which is in line with previous reports in pancreatic cancer cells [28]. We next tested AR-18 sensitivity against a panel of four additional pancreatic cancer cell lines. As shown in Fig. 1B, AR-18 potently reduced cell proliferation of all six pancreatic cancer cell lines tested in a dose- and time-dependent manner.

In order to determine whether GSK-3 is required for clonogenic survival of pancreatic cancer cells, exponentially growing PANC-1, and BxPC-3 cells were exposed to varying doses of AR-18 (10–50 μM) for 24 h. The number of colony-forming cells was reduced in a concentration-dependent manner by AR-18 (Fig 1C), and at 50 μM AR-18 the number of colony-forming PANC-1 and BxPC-3 cells were 0.055 ± 0.02 and 0.022 ± 0.006, respectively when compared with untreated controls.

### GSK-3 mediates NF-κB activation in pancreatic cancer cells

**Figure 2:**
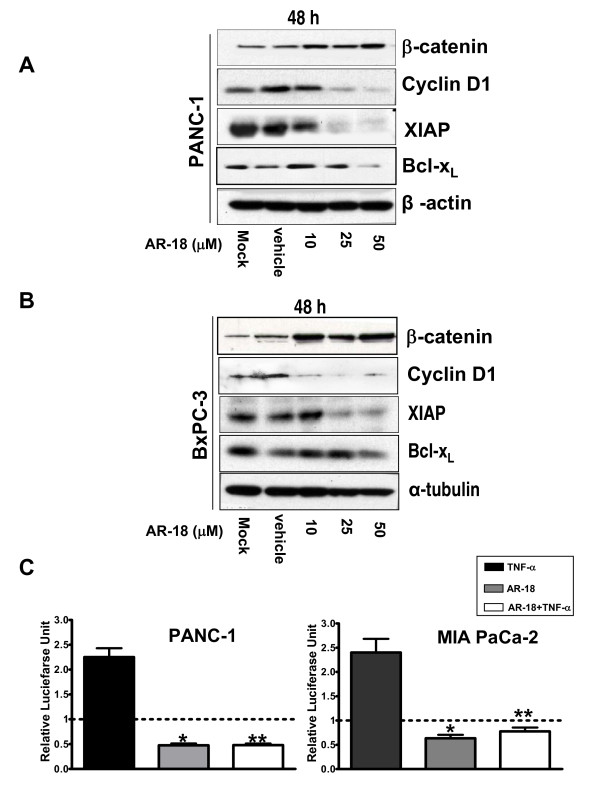
**Inhibition of GSK-3 disrupts NF-κB activity in pancreatic cancer cells in a dose-dependent manner**. **A-B**. Western blot analysis of expression of β-catenin and NF-κB target genes: XIAP, BcL-X_L_, and cyclin D1, in PANC-1 and BxPC-3 cell lines after exposure to AR-18 for 48 h. The change in the expression level of the proteins is compared against untreated or vehicle treated controls. Increase in cytosolic β-catenin level indicates GSK-3 inhibition. Both α-tubulin and β-actin were used as loading controls. **C**. Effect of GSK-3 disruption on basal and TNF-α induced NF-κB activity measured by luciferase reporter assay. PANC-1 and MIA PaCa-2 cells were exposed to AR-18 (50 μM, 8 h), TNF-α(30 ng/mL, 4 h), or both after co-transfection with TA-LUC NF-κB reporter and β-gal (internal control) constructs. The normalized values are relative to the untreated control (indicating basal level of NF-κB activity) which is represented by dotted line. Each column represents mean for at least four separate experiments, each with three replicates; error bars = ± SEM. (*) significant: (p < 0.0003) when compared to untreated control. (**) significant: (p < 0.0001) when compared to TNF-α treatment.

Recent evidence suggests that GSK-3 is a positive regulator of NF-κB [26-28,31]. To test this, we first treated PANC-1 and BxPC-3 cells with increasing concentrations of AR-18 for 48 h and examined effects on cytoplasmic β-catenin, which is negatively regulated by GSK-3 via the Wnt pathway. Inhibition of GSK-3 with increasing doses of AR-18 resulted in a dose-dependent increase in the levels of cytoplasmic β-catenin with ~twofold increase at 50 μM AR-18, when compared to control, which is the expected pharmacodynamic effect (Fig. 2A). We next examined the effects of AR-18 treatment on the expression of the NF-κB target genes XIAP, cyclin D1, and Bcl-X_L_, and found that expression of these proteins was also reduced significantly in a dose-dependent manner (Fig 2A–B). Similar results were obtained using the unrelated GSK3 inhibitor, LiCl (Additional file 1).

To test if GSK-3 inhibition could impact basal NF-κB activity in pancreatic cancer cells, PANC-1, MIA PaCa-2, PK-1 and PK-8 cells were transfected with TA-LUC NF-κB and treated with AR-18. In all cell lines AR-18 treatment (50 μM, 8 h) significantly decreased basal NF-κB activity when compared to untreated control (Fig. 2C, and data not shown).

Since TNF-α induced NF-κB activity was reported to be inhibited in MEFs genetically lacking the GSK-3β isoform [26], we tested this by treating PANC-1 and MIA PaCa-2 cells with TNF-α(30 ng/ml, 4 h) in the presence or absence of AR-18. In both cell lines, TNF-α induced NF-κB luciferase activity above background by ~2.5–fold in control cells, whereas in cells pretreated with AR-18 the levels of NF-κB luciferase remained lower than baseline, and were not significantly different from those seen with AR-18 alone (Fig. 2C). Together, these findings support the idea that GSK-3 positively regulates basal NF-κB activity [28] and that inhibition of GSK-3 abrogates the activation of NF-κB by TNF-α.

### Genetic knockdown of GSK-3 abolishes NF-κB activity in pancreatic cancer cells

**Figure 3:**
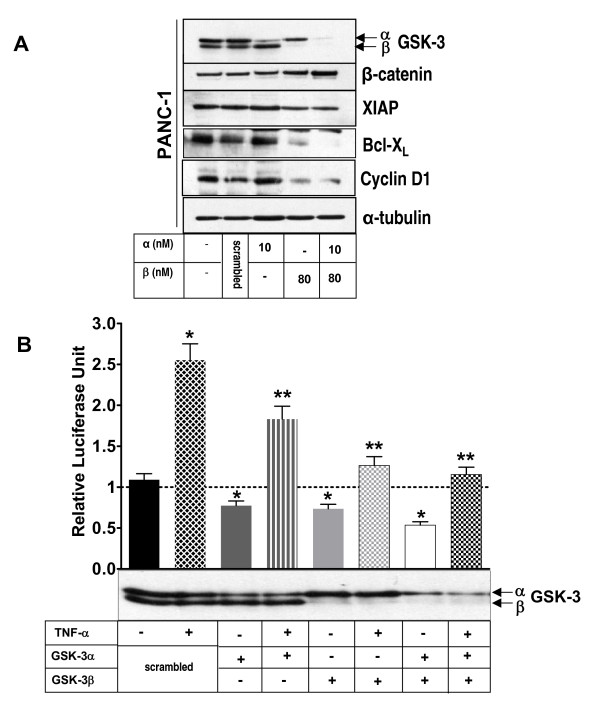
**Genetic knockdown of GSK-3 by siRNA results in disruption of NF-κB activity**. **A**. Western blot analysis of expression of NF-κB target genes XIAP, BcL-X_L_, and cyclin D1 in PANC-1 cells after transient knockdown of GSK-3 isoforms; α(10 nM siRNA), β (80 nM siRNA) or both. Expression level of total GSK-3 α or β isoforms confirms the genetic knockdown of the specified gene. Increased cytosolic β-catenin expression confirms GSK-3 inhibition. The change in the expression level of the proteins is compared against untreated or scrambled siRNA (negative control) treated controls. α-tubulin is used as loading control. **B**. Effect of genetic disruption of GSK-3 on basal and TNF-α induced NF-κB activity measured by luciferase reporter assay. PANC-1 cells were genetically knocked down for GSK-3 isforms α, β or both, and subsequently were co-transfected with TA-LUC NF-κB and β-gal (internal control) constructs. The cells were then exposed to TNF-α(30 ng/mL, 4 h). The normalized values are relative to the untreated control which is represented by dotted line (indicating basal level of NF-κB activity). Scrambled siRNA with or without TNF-α treatment is used as control. Each column represents mean for at least four experiments, each with three replicates; error bars = ± SEM. (*) significant: (p < 0.0005) when compared to untreated control. (**) significant: (p < 0.0001) when compared to TNF-α treatment. Western blot analysis of expression of GSK-3α and β isoforms in the above cells confirms successful knock down of the target genes.

Previous work suggests that inhibitors such as LiCl and AR-18 likely do not distinguish between the two GSK-3 isoforms [32]. To determine the effect of GSK-3 isoforms on NF-κB target gene expression in pancreatic cancer cells, we genetically depleted the expression of GSK-3α and GSK3β, alone or in combination, in PANC-1 cells using RNA interference. Following a 3-day exposure to GSK-3 specific siRNAs, immunoblotting showed >80% reduction in the expression levels of the corresponding GSK-3 isoforms when compared to untransfected or scrambled siRNA transfected controls (Fig. 3A). Depletion of either GSK-3α or β isoforms had minor effects on expression levels of Bcl-X_L_, XIAP, cyclin-D1, and β-catenin, with a greater effect shown by GSK-3β knockdown. However, consistent with pharmacological inhibition of GSK-3 using AR-18, simultaneous knockdown of both GSK-3 isoforms in PANC-1 cells led to a significantly greater effects on β-catenin, Bcl-X_L_, XIAP, and cyclin-D1 expression levels when compared to the single isoform knockdowns (Fig 3A).

To further test the effect of GSK-3 isoforms knockdown on basal NF-κB activity, we measured the level of NF-κB luciferase activity in knockdowns of PANC-1 cells. Inhibition of GSK-3α, β, or double knockdown of both GSK-3 isoforms significantly decreased the basal NF-κB activity (Fig. 3B); with greater effect exerted by genetic depletion of GSK-3β and the double knockdown (Fig. 3B). While TNF-α treatment induced >2.5 fold increase in non-specific (scrambled) siRNA treated cells, knockdown of either GSK-3 isoform resulted in a significant decrease in basal NF-κB luciferase activity and attenuated the effect of TNF-α, although these effects were greater with GSK3β knockdown. A large effect was seen when both isoforms were knocked down (Fig 3B), suggesting that whereas GSK3α is able to stimulate NF-κB activity, this is mediated principally by GSK-3β.

### GSK-3 inhibition does not enhance the anti-tumor effects of gemcitabine in pancreatic cancer *in vitro*

**Figure 4:**
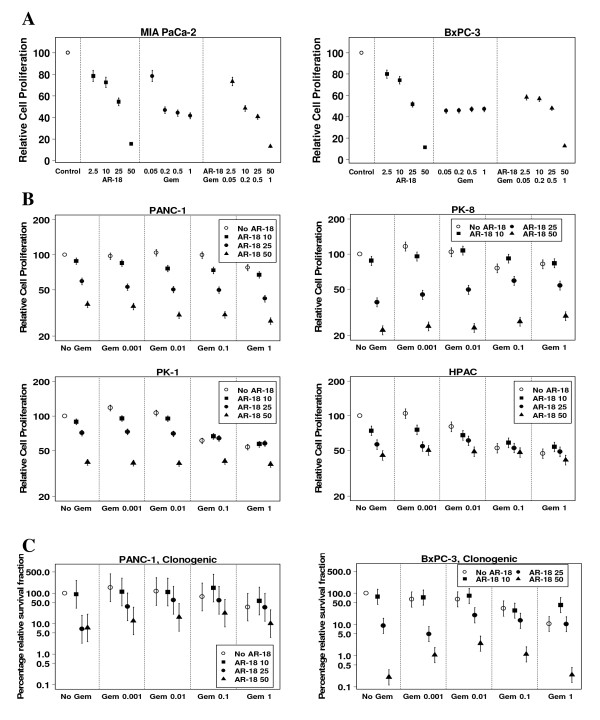
**Effects of AR-18 on gemcitabine sensitivity**. **A**. Growth inhibitory effect of AR-18 (2.5–50 μM), gemcitabine (0.05–1.0 μM), and their combination in a 200:1 AR-18 to Gemcitabine ratio was measured by the SRB proliferation assay in MIA PaCa-2 and BxPC-3 after 48 h of exposure. Each point represents mean from three experiments, each with six replicates; error bars = ± SEM. Gem: gemcitabine. The results are indicated by relative cell proliferation as a percentage of solvent control. **B**. Growth inhibitory effect of AR-18 (10–50 μM), gemcitabine (0.001–1.0 μM), and their combination was measured by the SRB proliferation assay in PANC-1, HPAC, PK-1, and PK-8 cell lines after 48 h of exposure. Each point represents mean from three separate experiments, each with six replicates; error bars = ± SEM. Gem: gemcitabine. The results are indicated by relative cell proliferation as a percentage of solvent control. **C**. Effect of AR-18 (10–50 μM), gemcitabine (0.001–1.0 μM), and their combination on colony-forming capacity of PANC-1 and BxPC-3 cells was measured by colonogenic assay. Control cells were given vehicle solution. Means for four separate experiments, each with three replicates; error bars = ± SEM. Gem: gemcitabine. The results are relative to vehicle treated control.

Using the SRB cell proliferation assay, the growth of BxPC-3 and MIA PaCa-2 cell lines was measured after 24, 48, and 72 h of exposure to a range of concentrations of either AR-18, gemcitabine, or a concurrent combination of both drugs; using either a fixed ratio of 200:1 AR-18 to gemcitabine, or variable doses of both drugs. AR-18 produced a steep dose-response over the 10–50 μM concentration range and this effect increased with the duration of exposure (Fig 4; 24 and 72 h data not shown). In contrast, the gemcitabine dose-response showed a plateau at low concentrations, and sensitivity was greatly influenced by the duration of drug exposure, consistent with the cell cycle phase-specificity of this agent.

Contrary to our hypothesis, combining both drugs either in a fixed ratio or variable doses was not synergistic against BxPC-3 or MIA PaCa-2 cells when compared to the single agents, across a wide range of concentrations and treatment times (Fig. 4A; 24 and 72 h data not shown). We also treated the four other pancreatic cancer cell lines using variable doses of both drugs for different time points. As seen in Fig 4B, with the exception of PANC-1 that showed a statistically-significant synergistic effect at some dose levels (Fig. 4B; 24 and 72 h data not shown), the drug combination was either sub-additive or even antagonistic. Because of the possibility that AR-18 might be antagonizing the effects of gemcitabine by reducing movement through S-phase, we also tested if prior exposure to gemcitabine sensitized to AR-18 but did not identify positive drug interaction under any of the conditions used.

To further investigate the interactions of AR-18 and gemcitabine, we tested the effect on the colony-forming capacity of PANC-1 and BxPC-3 cell lines. The cells were exposed to doses of AR-18, gemcitabine or their combination similar to those used for the SRB assay. No evidence of drug synergy was observed across a wide range of drug concentrations (Fig 4C).

**Figure 5:**
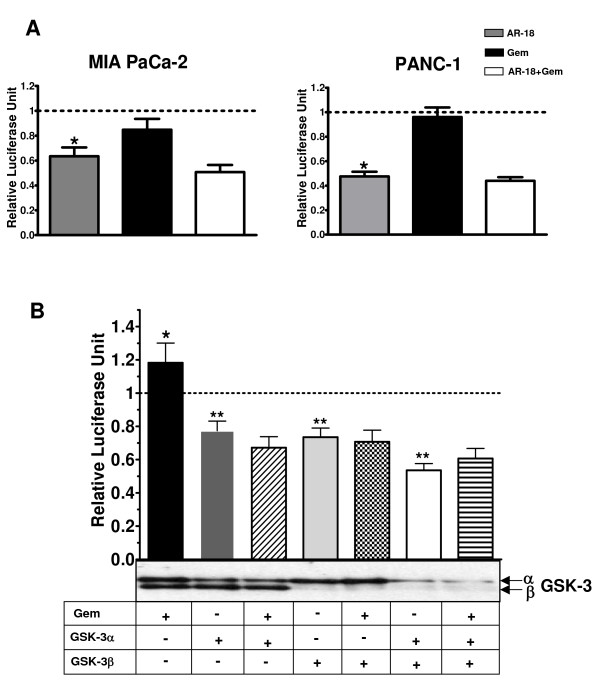
**Effects of gemcitabine combined with GSK-3 inhibition on NF-κB**. **A**. Effect of AR-18 (50 μM, 8 h), gemcitabine (10 μM, 8 h), and their combination measured by NF-κB luciferase reporter assay. PANC-1 and MIA PaCa-2 cells were co-transfected with TA-LUC NF-κB reporter construct and β-gal (internal control) and then exposed to AR-18, gemcitabine or both. The normalized values are relative to the untreated control which is represented by dotted line (indicating basal level of NF-κB activity). Each column represents the mean for at least four experiments, each with three replicates; error bars = ± SEM. (*) significant: (p < 0.0003) when compared to untreated control. Gem: gemcitabine. **B**. Effect of genetic disruption of GSK-3 and its combination with gemcitabine on NF-κB activity measured by luciferase reporter assay. PANC-1 cells were genetically knocked down for GSK-3 isforms α(10 nM), β (80 nM) or both, and subsequently were co-transfected with TA-LUC NF-κB and β-gal (internal control) constructs. The genetically treated or untreated cells were then exposed to gemcitabine (10 μM, 8 h). The normalized values are relative to the untreated control which is represented by dotted line (indicating basal level of NF-κB activity). Each column represents the mean for at least four experiments, each with three replicates; error bars = ± SEM. (*) significant: (p = 0.08) when compared to untreated control. (**) significant: (p < 0.0005) when compared to untreated control. Gem: gemcitabine. Western blot analysis of expression of GSK-3α and β isoforms in the above cells confirms successful knock down of the target genes.

Since treatment with gemcitabine was reported to cause NF-κB activation in pancreatic cancer cells *in vitro *[7], we tested if this effect is sensitive to GSK-3 inhibition. Consequently, TA-LUC NF-κB transfected PANC-1, MIA PaCa-2, PK-1, and PK-8 cells were exposed to gemcitabine (10 μM), AR-18 (50 μM), or both for 8 h and NF-κB activity was examined. We found a moderate increase in NF-κB activity effect in PANC-1 cells that appeared to be dependent on the experimental conditions (Fig. 5A and 5B). No significant increase was seen in MIA PaCa-2, PK1, and PK-8 cells (Fig. 5A, and data not shown). Although AR-18 significantly reduced basal NF-κB activity in all the cell lines, the combination of gemcitabine and AR-18 produced similar effects on the NF-κB reporter to those seen with single agent AR-18 (Fig. 5A, and data not shown). Furthermore, when we combined gemcitabine with transient knockdown of GSK-3 isoforms in PANC-1 cells, there was no increase in NF-κB activity (Fig. 5B).

### Similar to AR-18, curcumin inhibits NF-κB activity, but fails to sensitize pancreatic cancer cells to gemcitabine effect *in vitro*

**Figure 6:**
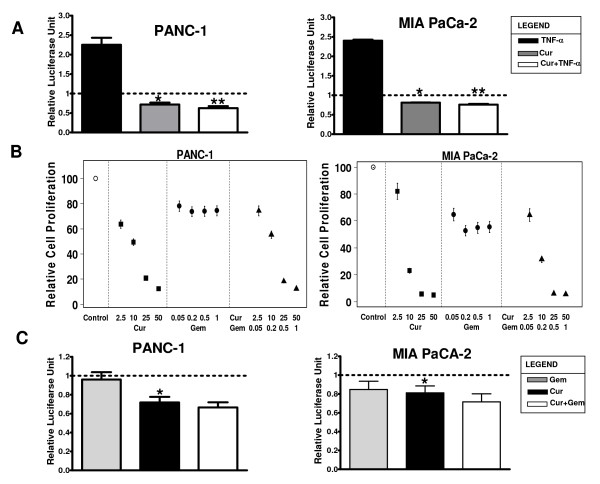
**NF-κB inhibition by curcumin does not increase sensitivity to gemcitabine in pancreatic cancer cells**. **A**. Effect of curcumin on basal and TNF-α induced NF-κB activity measured by luciferase reporter assay. PANC-1 and MIA PaCa-2 cells were exposed to curcumin (50 μM, 8 h), TNF-α(30 ng/mL, 4 h), or both after co-transfection with TA-LUC NF-κB reporter and β-gal (internal control) constructs. The normalized values are relative to the untreated control which is represented by dotted line (indicating basal level of NF-κB activity). Each column represents the mean for at least four separate experiments, each with three replicates; error bars = ± SEM. (*) significant: (p < 0.001) when compared to untreated control. (**) significant: (p < 0.0001) when compared to TNF-α treatment. Cur: curcumin, Gem:gemcitabine. **B**. Growth inhibitory effect of curcumin (2.5–50 μM), gemcitabine (0.05–1.0 μM), and their combination in a 200:1 (curcumin to gemcitabine) ratio was measured by the SRB proliferation assay in MIA PaCa-2 and PANC-1 after 48 h of exposure. Each point represents the mean from three separate experiments, each with six replicates; error bars = ± SEM. Cur: curcumin, Gem: gemcitabine. The results are indicated by relative cell proliferation as a percentage of solvent control. **C**. Effect of curcumin (50 μM, 8 h), gemcitabine (10 μM, 8 h), and their combination measured by luciferase reporter assay. PANC-1 and MIA PaCa-2 cells were co-transfected with TA-LUC NF-κB reporter construct and β-gal (internal control) and then exposed to curcumin, gemcitabine or both. The normalized values are relative to the untreated control which is represented by dotted line (indicating basal level of NF-κB activity). Each column shows the mean for at least four experiments, each with three replicates; error bars = ± SEM. (*) significant: (p < 0.001) when compared to untreated control. Cur: curcumin, Gem: gemcitabine.

Since GSK-3 could have both pro- and anti-apoptosis effects, we considered that the lack of sensitization to gemcitabine using AR-18 might be explained by the effect of GSK-3 on targets other than NF-κB that could potentially modify chemotherapy sensitivity. To address this, we compared the effects using curcumin, which inhibits NF-κB through different mechanisms. Similar to previous reports and consistent with our observations using AR-18, both PANC-1 and MIA PaCa-2 cells showed a significant decrease in basal as well as TNF-α induced NF-κB activity after exposure to curcumin (50 μM) for 8 h (Fig. 6A). We then tested for synergism by exposing PANC-1 and MIA PaCa-2 cells to various doses of curcumin, gemcitabine, or their combination in doses similar to those used by Kunnumakkara *et al. *[20]. Consistent with their findings, 48 h exposure to curcumin had a significant growth inhibitory effect on these cell lines measured by SRB assay (Fig. 6B). However, as seen in Fig 6B and similar to our results using AR-18, NF-κB inhibition by curcumin did not sensitize the pancreatic cancer cells to gemcitabine. Likewise, the effect of curcumin down-regulating NF-κB luciferase activity was not significantly altered by combined treatment with gemcitabine (Fig. 6C).

## Discussion

Chemotherapy resistance of pancreatic cancer has been previously associated with hyperactivity of NF-κB [7,11,19,33]. The discovery that GSK-3 regulates NF-κB [26], and that its inhibition has anti-inflammatory and growth inhibitory effects, holds promise to resolve the problem of drug resistance in cancers with inflammatory origin including pancreatic cancer [26,28,34]. In this paper, using a panel of six genetically distinct pancreatic cancer cell lines we confirmed previous reports that pharmacological inhibition of GSK-3 suppresses NF-κB transcriptional activity and is toxic to pancreatic cancer cells in a dose- and time-dependent manner [28]. We also show for the first time that GSK-3 inhibition potently reduces the clonogenic survival of pancreatic cancer cells. However, contrary to our hypothesis GSK-3/NF-κB inhibition did not sensitize to gemcitabine chemotherapy.

GSK-3 is a kinase involved in many cellular processes including energy metabolism, transcriptional regulation, cell adhesion, and protein turnover [35,36]. This complexity of action results in a potential for GSK-3 to exert both pro- and anti-apoptotic effects that appears to be cell- and context-dependent [37]. The anti-apoptotic activity of GSK-3 has been attributed in part to the stimulation of NF-κB activity through an unknown mechanism, as shown by this study and others [26,28,31]. It has been previously shown that β-catenin has inhibitory effects on NF-κB [38], which could explain the effects of GSK-3 inhibition since this results in the accumulation of β-catenin. Although the accumulation of β-catenin could potentially be cancer-promoting, this did not rescue pancreatic cells from death due to lack of NF-κB activity, further supporting the importance of NF-κB activity in maintaining the survival of these cells, whereas Wnt/β-catenin appears to play a less prominent role in pancreatic cancer development [34]. The above findings in conjunction with others [26,28,39] lend support to a positive role for GSK-3 activity in the regulation of NF-κB, rather than inhibition through S468 phosphorylation as has been described in other systems [23].

Since GSK-3 inhibitors including Wnt, LiCl, and AR-18 likely do not discriminate between GSK-3 α and β isoforms [26,40], and given that functional redundancy of GSK-3 isoforms in the context of Wnt/β-catenin signaling has been previously described in mouse embryonic stem cells [22], we investigated whether NF-κB regulation by GSK-3 was isoform-specific in pancreatic cancer cells. Transient genetic knockdown of GSK-3α had a minor impact on β-catenin, cyclin D1 and XIAP expression when compared to GSK-3β knockdown, whereas GSK-3α/β double knockdown demonstrated the greatest effect. Similarly, transient knockdown of either GSK-3α or GSK-3β significantly reduced both the basal as well as the TNF-α induced NF-κB activity of PANC-1 cells, although GSK-3β knockdown exerted the greater effect and the double knockdown of both isoforms was the most effective. Collectively, these findings suggest that although NF-κB activity in pancreatic cancer is responsive to both GSK-3 isoforms, GSK-3β is the major regulator. Our finding of the differential effects of GSK-3 isoforms is in agreement with the previously proposed functional redundancy of GSK-3 isoforms [22], and also confirms the importance of GSK-3β in NF-κB regulation [26,41]. Furthermore, our observations also raise the possibility of NF-κB cross-regulation by GSK-3 isoforms in pancreatic cancer. This phenomenon could have important implications with regards to the development of isoform specific GSK-3 inhibitors, and further work in this area appears indicated.

Since GSK-3 inhibition efficiently suppresses NF-κB in pancreatic cancer cells, and downregulates NF-κB targets associated with chemotherapy resistance such as XIAP and Bcl-X_L_, it seemed reasonable to predict that this would also sensitize these cells to gemcitabine. In all six cell lines tested, treatment with AR-18 as a single agent was growth inhibitory in a dose- and time-dependent manner, similar to a previous report [28]. However, with the exception of PANC-1 the combination with gemcitabine was not synergistic. In fact, across a wide range of conditions and drug combinations, the interactions ranged from additive to antagonistic effects. Similar additive or antagonistic effects were observed in PANC-1 or BxPC-3 cells using clonogenic survival as the endpoint.

The lack of sensitization to gemcitabine by GSK-3 inhibition in pancreatic cancer might be due to number of reasons: 1) Some of the proteins targeted by GSK-3 for proteasomal degradation, including Mcl-1, β-catenin, and cdc25 [42-44], have cancer-promoting effects. Consequently, GSK-3 inhibition might have adverse effects by stabilizing these proteins; 2) GSK-3 inhibition is reported to confer resistance to chemotherapy through suppression of death receptor-mediated apoptosis [45,46]; 3) Gemcitabine treatment has been previously reported to induce NF-κB activity *in vitro *[7], and this effect might counteract the inhibition of NF-κB seen following treatment with AR-18. However, this appears not to be the case under the experimental conditions used, since in the present study the increase in NF-κB activity following gemcitabine exposure was modest and cell line dependent, and effectively inhibited by AR-18 or GSK-3 knockdown; 4) It is also possible that although NF-κB is hyper-activated in pancreatic cancer, it does not play a major role in gemcitabine resistance, which is in line with some recent reports [47-49], 5) We also considered that as gemcitabine cytotoxicity is cell cycle dependent, GSK-3 inhibition might antagonize gemcitabine by slowing entry into S-phase or causing cell cycle arrest. However, DNA content analysis by flow cytometry showed that the cell cycle effects of AR-18 were relatively modest, which is in line with a previous report that in contrast to some other GSK-3 inhibitors, AR-18 is a relative weak inhibitor of cyclin-dependent kinases [50]. Furthermore, we did not observe increased sensitization to the drug combination when cells were pre-exposed to gemcitabine prior to the addition of AR-18 (data not shown). Although the enhancement of gemcitabine toxicity following GSK-3 inhibition appears to be modest *in vitro*, we recognize that this does not exclude the potential for positive drug interaction *in vivo*, and this remains to be tested.

To investigate whether NF-κB inhibition by agents other than GSK-3 inhibitors could potentiate gemcitabine sensitivity, we tested the natural product curcumin. We found that curcumin inhibited both constitutive and TNF-α-induced NF-κB activity in PANC-1 and MIA PaCa-2 cells, and was also toxic in a dose-and time-dependent manner, which was consistent with previous reports [20,51]. However, in contrast to findings by Kunnumakkara *et al.*, and similar to our findings with AR-18, we did not observe enhancement of gemcitabine toxicity by curcumin. Thus, our findings do not support a role for NF-κB activity as a significant mediator of gemcitabine resistance in pancreatic cancer, or the corollary that NF-κB inhibition is able to overcome chemotherapy resistance. These results are corroborated by a recent study in colon cancer cell lines, where p65 overexpression could sensitize the cells to curcumin effects [47], and by other reports suggesting that NF-κB might function as a pro-apoptotic or a tumor suppressor factor [47-49,52], depending on the nature of apoptotic stimuli or the cell type.

Thus, although this work supports a model in which activated NF-κB is maintained by GSK-3 and promotes the survival of pancreatic cancer cells, we suggest that the major mechanisms of gemcitabine resistance are not dependent on NF-κB. Alternative mechanisms include alterations in drug uptake and metabolism, enhanced DNA repair proficiency, or activation of survival by other signaling pathways such as PI3-kinase/Akt. The exact role of GSK-3 in the maintenance of pancreatic cancer, the differential role of its isoforms in regulating NF-κB activity in these cells, and the mechanisms or conditions through which it maintains NF-κB activity remain unclear, although recent work suggests an important role for GSK-3 in the phosphorylation of IKK [39]. Furthermore, the mechanisms of cell death following GSK-3 inhibition appear not to be through classical apoptosis pathways as we did not observe PARP cleavage or loss of mitochondrial membrane potential, and AR-18 treated cells could not be rescued using the general caspase inhibitor zVAD (fmk) (data not shown). In summary, this work supports a potentially important role for GSK-3 inhibition in the treatment of pancreatic cancer, but cautions that further work examining the underlying mechanisms is needed for this to be rationally exploited in the clinic.

## Conclusion

Our observations suggest that although GSK-3 inhibition does not significantly sensitize to the standard chemotherapy agent gemcitabine, yet it is a promising new approach to the treatment of pancreatic cancer through disruption of NF-κB. We also conclude that NF-κB is not a key player in gemcitabine resistance of pancreatic cancer. Further work is needed to understand the mechanisms of the anticancer effect of GSK-3 inhibition, including the potential for rational combination with other targeted agents for the treatment of pancreatic cancer.

## Competing interests

The authors declare that they have no competing interests.

## Authors' contributions

SM was responsible for the study design, experimental work, data evaluation and analysis, and drafting the manuscript. SP was consulted extensively in the experimental design and interpretation of results, as well as in the preparation of the manuscript. DWH was the research supervisor, participated in the study design, assessment of the results, as well as drafting the manuscript.

## Pre-publication history

The pre-publication history for this paper can be accessed here:

http://www.biomedcentral.com/1471-2407/9/132/prepub

## Supplementary Material

Additional file 1 **Downregulation of NF-κB target gene expression upon LiCl treatment**. Western blot analysis of expression of NF-κB target genes XIAP, and cyclin D1in PANC-1 cells after exposure to LiCl (10–50 mM) for 48 h. KCl (10 mM) is used as vehicle control. Increased cytosolic β-catenin expression confirms GSK-3 inhibition in a dose-dependent manner. β-actin is used as loading control. Click here for file
