# Supplementary figures and images for: Glycogen synthase kinase-3 inhibition disrupts nuclear factor-kappaB activity in pancreatic cancer, but fails to sensitize to gemcitabine chemotherapy
Source: BMC Cancer. 2009 Apr 30;9:132. doi: 10.1186/1471-2407-9-132 (PMC2685435; doi:10.1186/1471-2407-9-132)

## Slide 1
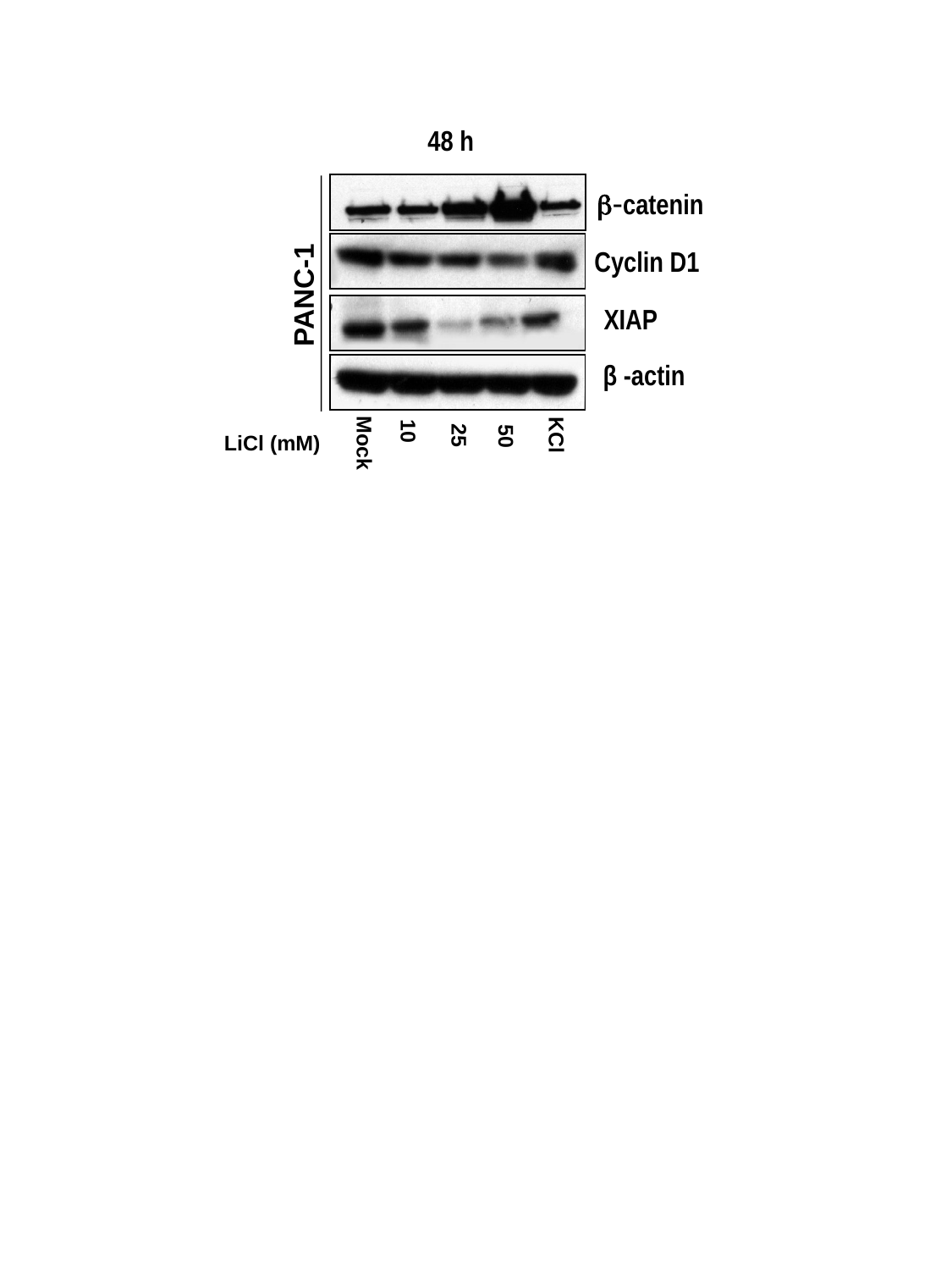

48 h
β-catenin
PANC-1
Cyclin D1
XIAP
β -actin
Mock
KCl
10
25
50
LiCl (mM)

Supplement: Additional file 1 — Downregulation of NF-κB target gene expression upon LiCl treatment. Western blot analysis of expression of NF-κB target genes XIAP, and cyclin D1in PANC-1 cells after exposure to LiCl (10–50 mM) for 48 h. KCl (10 mM) is used as vehicle control. Increased cytosolic β-catenin expression confirms GSK-3 inhibition in a dose-dependent manner. β-actin is used as loading control. [file 1471-2407-9-132-S1.ppt]
